# Amplified Detection of Iron Ion Based on Plasmon Enhanced Fluorescence and Subsequently Fluorescence Quenching

**DOI:** 10.1007/s40820-014-0005-5

**Published:** 2014-09-17

**Authors:** Lin Zhou, Han Zhang, Yanping Luan, Si Cheng, Li-Juan Fan

**Affiliations:** Jiangsu Key Laboratory of Advanced Functional Polymer Design and Application, College of Chemistry, Chemical Engineering and Materials Science, Soochow University, 199 Ren-Ai Road, Suzhou, 215123 Jiangsu People’s Republic of China

**Keywords:** Gold–silver alloy, Plasmon enhanced fluorescence, Fluorescence quenching, Iron ion

## Abstract

A facile and rapid approach for detecting low concentration of iron ion (Fe^3+^) with improved sensitivity was developed on the basis of plasmon enhanced fluorescence and subsequently amplified fluorescence quenching. Au_1_Ag_4_@SiO_2_ nanoparticles were synthesized and dispersed into fluorescein isothiocyanate (FITC) solution. The fluorescence of the FITC solution was improved due to plasmon enhanced fluorescence. However, efficient fluorescence quenching of the FITC/Au_1_Ag_4_@SiO_2_ solution was subsequently achieved when Fe^3+^, with a concentration ranging from 17 nM to 3.4 μM, was added into the FITC/Au_1_Ag_4_@SiO_2_ solution, whereas almost no fluorescence quenching was observed for pure FITC solution under the same condition. FITC/Au_1_Ag_4_@SiO_2_ solution shows a better sensitivity for detecting low concentration of Fe^3+^ compared to pure FITC solution. The quantized limit of detection toward Fe^3+^ was improved from 4.6 μM for pure FITC solution to 20 nM for FITC/Au_1_Ag_4_@SiO_2_ solution.

## Introduction

Noble metal nanoparticles (NPs), such as Au and Ag, are known to dramatically change the optical properties of nearby fluorophores due to the localized surface plasmon resonance (LSPR) at the surface of the metal NPs [1]. When fluorescent molecules are localized adjacent to metal surface, their fluorescence emission intensity can be altered enormously, forming the basis of plasmon enhanced fluorescence (PEF) [2]. However, if fluorescent molecules are directly in contact with the metal surface, fluorescence quenching would be suffered due to the non-radiative energy and/or charge transfer from molecules to the metal. A nanometer-thin spacer layer, made of either polyelectrolytes or silica, is usually employed to separate the molecules away from the metal surface to avoid fluorescence quenching [2–4]. The fluorescence of fluorophores can make a continuous transition from fluorescence quenching to fluorescence enhancement with increasing thickness of the silica layer [5–9]. Great attentions have been paid to prepare metal core-silica/fluorophore-shell nanostructure for various applications, such as optical property [7], cellular imaging [10], and photothermal therapy [11]. The excited surface plasmon can deliver significant control over the optical field and enhance the light absorption or fluorescence emission of molecule, which is critical to improving the sensitivity of fluorescence spectroscopy [12].

During the past couple of decades, fluorescence detection offers a promising approach for simple and rapid tracking of heavy metal ions [13, 14]. Contamination of water by metal ions (*e.g.*, mercury, copper, iron) can cause serious environmental and health problems because of acute and/or chronic toxicity to biological organisms [15–17]. Therefore, monitoring of metal ion levels in water is very important for the environment or our health. The conventional detection concentration of metal ion based on fluorophore or conjugated polymer is on the micromolar (μM) level [12, 18]. Therefore, many AuNPs-based colorimetric, fluorescent or refractive index sensor have been developed to detect lower concentration of metal ions through utilizing fluorescent noble metal nanoclusters or metal NPs fluorescently labeled DNA nanohybrids structures [9, 15–17, 19–23]. However, complicated processes together with expensive fluorophores or coupling reagents for labeling the probes and/or target analytes were involved in the preparation of fluorophore conjugated AuNPs probes [24]. Specialized synthetic skills and complicated purification procedures are rather time-consuming and disadvantage for further practical application. Thus, developing a facile, rapid, and label-free strategy for the detection of metal ions is still highly desired.

**Fig. 1:**
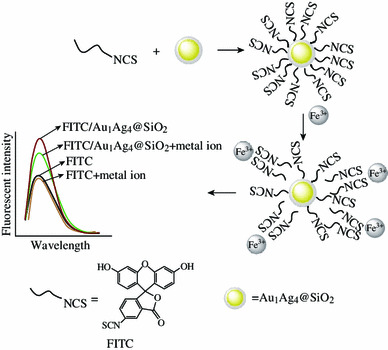
Schematic illustration of the detection of Fe^3+^ on the basis of fluorescence enhancement and subsequently fluorescence quenching

Fluorescein isothiocyanate (FITC) has many reactive groups, such as isothiocyanate group (–N=C=S), carboxylic group, hydroxyl group, and carbonyl group (Fig. 1). It has been widely used as a fluorescent label to attach to proteins via the amine group or been tailored for various chemical and biological applications due to different attachment groups, high fluorescence intensity, and great photostability [24–26]. FITC molecules can be readily attached to the surface of AuNPs through their isothiocyanate group [25, 27] and the isothiocyanate group of FITC can also be bond with iron ion (Fe^3+^) to form metal-isothiocyanate complexes [28].

Herein, we developed a facile and rapid approach to detecting low concentration of Fe^3+^ with improved sensitivity on the basis of plasmon enhanced fluorescence and subsequently amplified fluorescence quenching. First, the fluorescence of the FITC solution was greatly improved by the addition of Au_1_Ag_4_@SiO_2_ NPs. And then, the fluorescence of solution was dramatically quenched again when Fe^3+^ was added. The extent of fluorescence quenching for the FITC/Au_1_Ag_4_@SiO_2_ solution toward Fe^3+^ is apparently larger than that for pure FITC solution, which resulted in the improved sensitivity for detecting low concentration of Fe^3+^.

## Experimental Methods

Au_1_Ag_4_ alloy NPs were synthesized by reducing HAuCl_4_ and AgNO_3_ solution simultaneously with sodium citrate, according to a reported procedure with slight modifications [30]. 6 mL of HAuCl_4_ aqueous solution (0.01 mol/L) and 24 mL of AgNO_3_ aqueous solution (0.01 mol/L) were added to 70 mL ultra-filtered water. The mixture was heated to boiling, and then 6 mL of sodium citrate aqueous solution (1 wt%) was injected quickly. The mixture was further boiled for about 60 min and the color of the solution turned from colorless to yellow within 10 min. Subsequently, 6 mL of sodium citrate aqueous solution was injected again. The mixture was kept boiling for 60 min and then cooled to room temperature.

100 mL of as-synthesized Au_1_Ag_4_ alloy NPs was centrifuged at 6,500 rpm, and then was redispersed into 3 mL of water. 1 mL of concentrated Au_1_Ag_4_ alloy NPs were added into 30 mL of iso-propanol/9 mL of deionized water mixture with vigorous stirring. Different volume of TEOS (10 mM in iso-propanol) was added to the reaction mixture immediately followed by the addition of 0.75 mL of 25 % ammonium hydroxide. The Au_1_Ag_4_@SiO_2_ NPs with different silica thicknesses were made by tuning the volume of TEOS to 1.6, 3.2, 4.8, and 6.0 mL. The reaction solution was kept at 30 °C for 24 h under stirring.

In a typical process, 400 μL Au_1_Ag_4_@SiO_2_ solution was added to 2.5 mL FITC aqueous solution (5 × 10^−6^ M), then different volumes of Fe^3+^ solutions were added. In order to eliminate the possibility of contaminations, Fe^3+^ solutions were prepared by dissolving desired amount of solid FeCl_3_ (AR) into ultra-filtered water. The mixture was obtained by repeated gentle inversion for 10 s and then was directly measured for fluorescence emission spectra. Ultra-filtered water was used in all experiments.

Transmission electron microscope (TEM) was taken with TecnaiG220, FEI company microscope operated at 200 kV. Ultraviolet–visible spectroscopy (UV-Vis) extinction spectra were measured with Shimadzu UV-3150 spectrophotometer. Fluorescence emission measurements were recorded with a Horiba FluoroMax-4 spectrofluorometer. Fluorescence lifetimes were performed on FL3-TCSPC Fluorescence Spectroscopy (Horiba Jobin–Yvon Inc., France).

## Results and Discussion

Our strategy for fluorescence enhancement was to disperse as-synthesized Au_1_Ag_4_@SiO_2_ NPs into FITC solution. The fluorescence of the FITC solution was improved with the addition of Au_1_Ag_4_@SiO_2_ NPs due to PEF. However, the field enhancement region of metal NPs are confined in close vicinity to the surface of plasmonic metal NPs and the fluorescence enhancement is very sensitive to the surrounding environment of metal NPs [2], such as the space between fluorescent molecule and metal NPs. Thus, the previous enhanced fluorescence of FITC by the Au_1_Ag_4_@SiO_2_ NPs was dramatically quenched again when Fe^3+^ was dropped into the FITC/Au_1_Ag_4_@SiO_2_ solution. The fluorescence quenching is possibly attributed to the complexation action between the isothiocyanate group of FITC and Fe^3+^, which resulted that FITC molecule was away from the field enhancement region of Au_1_Ag_4_@SiO_2_ NPs (Fig. 1).

**Fig. 2:**
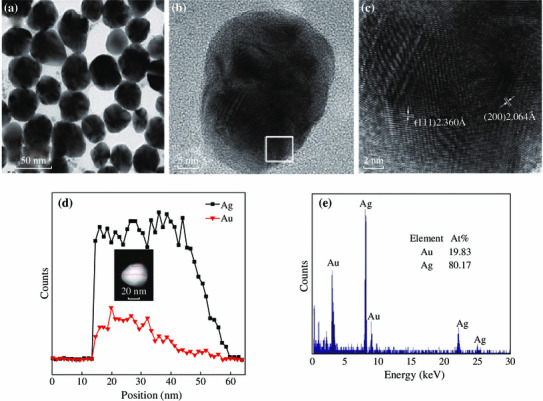
**a** TEM image of Au_1_Ag_4_ alloy NPs. **b** and **c** High-resolution electron images of Au_1_Ag_4_ NPs. **d** Cross-sectional compositional STEM-EDS line scan profiles recorded from one nanoparticle. Inset is HAADF-STEM image. **e** TEM-EDS spectrum of a single Au_1_Ag_4_ NPs

Note that the local electric field enhancement is dependent on the plasmon resonance wavelength of metal NPs. The fluorescence intensity reaches to the maximum when the plasmon wavelength of the metal NPs is between the absorption and emission peak of the fluorescent molecules [29]. However, localized plasmonic resonances have certain peak widths due to damping; the maximal effect is also believed to occur when the emission or excitation peak overlaps closely with the plasmon resonance peak [2]. Only when a plasmon resonance is excited at its peak wavelength can the maximal field enhancement be obtained [2]. We synthesized Au_1_Ag_4_ NPs with an extinction peak at 437 nm by the co-reduction of chlorauric acid (HAuCl_4_) and silver nitrate (AgNO_3_) solution according to a reported procedure with slight modifications [30]. The average diameter of Au_1_Ag_4_ NPs is 35 ± 5 nm. The High-resolution TEM (HRTEM) images of the Au_1_Ag_4_ NPs showed the d-spacing for lattice fringes and the corresponding selected area matched well with that of the (111) and (200) planes of face-centered cubic Au and Ag (Fig. 2). High-angle annular dark-field (HAADF) scanning transmission electron microcopy (STEM) image with energy dispersive X-ray spectroscopic (EDX) elemental line profiling and TEM-EDS (TEM-energy dispersive spectrum) of single NPs revealed the alloy structure and composition of NPs (Fig. 2).

**Fig. 3:**
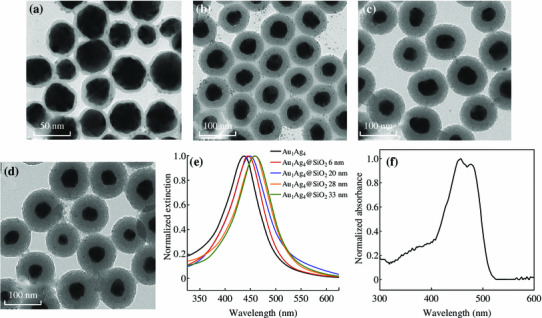
TEM images of Au_1_Ag_4_@SiO_2_ with different thicknesses of silica shells: **a** 6 nm; **b** 20 nm; **c** 28 nm; **d** 33 nm. **e** The corresponding extinction spectra. **f** UV–Vis spectrum of the FITC solution

A SiO_2_ shell with variable thicknesses was coated on the surface of Au_1_Ag_4_ NPs by a sol–gel reaction of TEOS according to the reported process [24]. The fluorescent molecule–metal distance was controlled by varying the thickness of the SiO_2_ spacer shell. The surface plasmon resonance peak of Au_1_Ag_4_@SiO_2_ NPs red-shifted from 445 to 458 nm as the thickness of the silica shell increased from 6 nm to 33 nm (Fig. 3a–e). The extinction peak of Au_1_Ag_4_@SiO_2_ NPs is close to the absorption peak of the FITC solution (454–474 nm) (Fig. 3f). The silica layer not only provided a distance-dependent PEF but also offered the robustness, chemical inertness, and the versatility needed for the conjugation of fluorescent molecule [4, 7, 31].

**Fig. 4:**
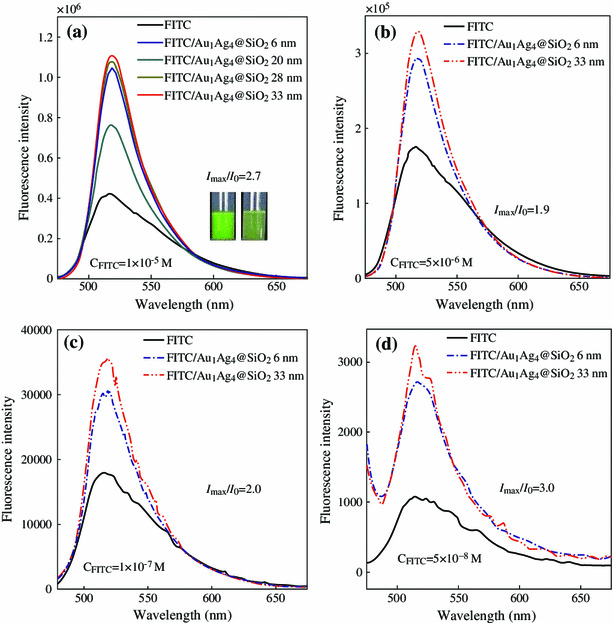
Fluorescence emission spectra of the FITC solution with the addition of Au_1_Ag_4_@SiO_2_ NPs. The concentration of the FITC solution is: **a** 1 × 10^−5^ M; **b** 5 × 10^−6^ M; **c** 1 × 10^−7^ M and **d** 5 × 10^−8^ M, respectively. The volume of the FITC solution is 2.5 mL. The excitation wavelength is 450 nm

Subsequently, Au_1_Ag_4_@SiO_2_ NPs were directly dispersed into the FITC solution and the influence of Au_1_Ag_4_@SiO_2_ NPs on the fluorescence of the FITC (1 × 10^−5^ M) were investigated. The emission spectra of the FITC/Au_1_Ag_4_@SiO_2_ solution as well as pure FITC solution are presented in Fig. 4a. The fluorescence of the FITC solution increased greatly as the thickness of the silica shell varied. Maximal fluorescence intensity (about 2.7 times) was observed when the thickness of silica shell was 33 nm. Enhanced fluorescence is also easily discernible to the naked eye under 365 nm irradiation (the photos inset in Fig. 4a). The fluorescence enhancement can also be achieved when decreasing the concentration of FITC aqueous solution. The max fluorescence intensity of the FITC/Au_1_Ag_4_@SiO_2_ solution is 1.9, 2.0, and 3.0 folds of pure FITC solution, corresponding to the FITC solution with a concentration of 5 × 10^−6^, 1 × 10^−7^, and 5 × 10^−8^ M, respectively (Fig. 4b–d).

**Fig. 5:**
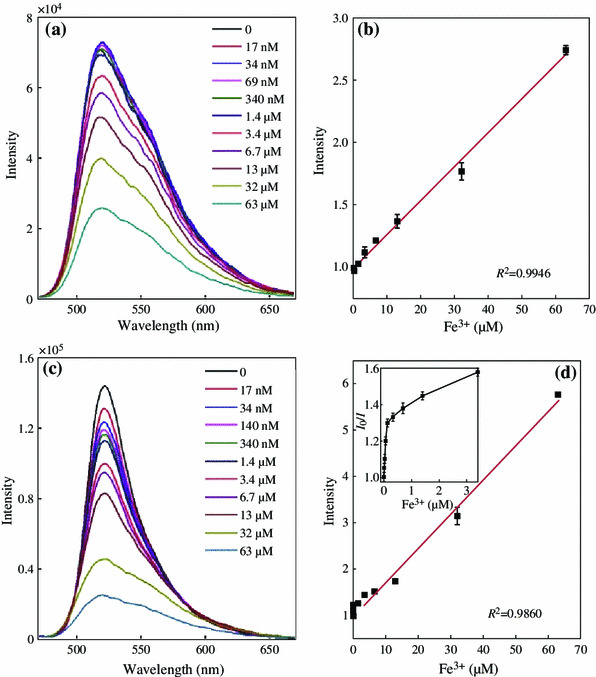
Fluorescence spectra of **a** FITC and **c** FITC/Au_1_Ag_4_@SiO_2_ in the presence of Fe^3+^. **b** and **d** The corresponding relationship between intensity ratio (*I*_0_/*I*) and Fe^3+^ concentration. The excitation wavelength is 450 nm

**Fig. 6:**
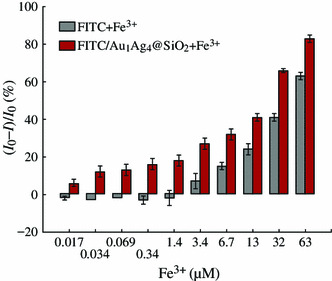
Relative fluorescence intensity ((*I*_0_ − *I*)/*I*_0_) of the FITC solution and FITC/Au_1_Ag_4_@SiO_2_ solution toward Fe^3+^ with a concentration from 17 nM to 63 μM

In general, the field-enhanced fluorescence is very sensitive to the surrounding environment of metal NPs. Note that the fluorescence of the solution was varied as the thickness of silica shell changed, we further investigated the metal ion-responsive property of the FITC/Au_1_Ag_4_@SiO_2_ solution. The fluorescence response of the FITC and FITC/Au_1_Ag_4_@SiO_2_ solution toward Fe^3+^ with a concentration ranging from 17 nM to 63 μM were compared and their fluorescence spectra were recorded (Fig. 5). We found that 34 nM Fe^3+^ was enough to show efficient fluorescent quenching (14 %) of the FITC/Au_1_Ag_4_@SiO_2_ solution, whereas no fluorescence quenching was observed in pure FITC solution. An apparent fluorescence quenching of pure FITC solution was found until Fe^3+^ concentration reached 3.4 μM. The fluorescence intensity of the solution kept decreasing as the concentration of Fe^3+^ increased. About 32 and 68 % of quenching toward 3.4 and 32 μM of Fe^3+^ were achieved for FITC/Au_1_Ag_4_@SiO_2_ solution, but only about 11 and 43 % of quenching were observed in pure FITC solution under the same condition (Fig. 6). Relative fluorescence intensity ((*I*_0_ − *I*)/*I*_0_) of the FITC solution and FITC/Au_1_Ag_4_@SiO_2_ solution toward Fe^3+^ with a concentration ranging from 17 nM to 63 μM was shown in Fig. 6. The relative fluorescence intensity displayed in Fig. 6, indicated that the extent of fluorescence quenching of the FITC/Au_1_Ag_4_@SiO_2_ solution upon Fe^3+^ was apparently larger than that in FITC solution and the sensitivity of detecting low concentration of Fe^3+^ was dramatically improved.

The intensity ratio *I*_0_/*I* of pure FITC solution and FITC/Au_1_Ag_4_@SiO_2_ solution displayed a good linear relationship versus Fe^3+^ concentration ranging from 3.4 to 63 μM, which was easily described by the Stern–Volmer equation (*I*_0_/*I* = 1 + *K*_sv_[*Q*]), where *I*_0_ and *I* are the fluorescence intensity of the FITC solution in the absence and presence of metal ion, *K*_sv_ is the Stern–Volmer fluorescence quenching constant, and *Q* is the concentration of metal ion. The resulting linear equations were *I*_0_/*I* = 1 + 0.027[Fe^3+^] and *I*_0_/*I* = 1 + 0.074[Fe^3+^], corresponding to FITC solution and FITC/Au_1_Ag_4_@SiO_2_ solution, respectively. However, the intensity ratio *I*_0_/*I* for FITC/Au_1_Ag_4_@SiO_2_ solution displayed a different response versus lower concentration of Fe^3+^ ranging from 17 nM to 3.4 μM. A quantitative analysis of Fe^3+^ at the lower concentration was achievable (*I*_0_/*I* = 1 + 2.84[Fe^3+^], *R*^2^ = 0.9498). The quantized limit of detection (LOD) toward Fe^3+^ was calculated to be 20 nM, whereas the LOD value for pure FITC solution toward Fe^3+^ is only 4.6 μM.

**Fig. 7:**
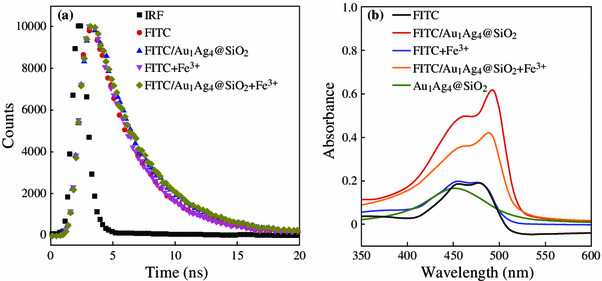
**a** The fluorescence intensity decays of FITC and FITC/Au_1_Ag_4_@SiO_2_ solution without or with Fe^3+^. The instrument response function (IRF) is also included. The concentration of Fe^3+^ is 3.4 μM. **b** Absorption spectra of the FITC solution, Au_1_Ag_4_ NPs and the FITC solution in the absence or presence of Au_1_Ag_4_@SiO_2_ NPs or Fe^3+^. The concentration of Fe^3+^ is 340 nM

We preliminary investigated the possible reasons for fluorescence enhancement and subsequently fluorescence quenching in this system. Usually, the PEF arises from two contributions. One is the increase in the radiative emission rate, which leads to very short fluorescence lifetime. The other is the increase in the excitation rate due to the local electric field enhancement near the surfaces of metal NPs, which result in surface plasmon enhanced absorption [1, 32, 33]. Figure 7a shows the time-resolved measurements on the emission dynamics of the FITC solution without and in the presence of Au_1_Ag_4_@SiO_2_ NPs. No notable decrease of the fluorescence lifetimes were observed for pure FITC solution and FITC/Au_1_Ag_4_@SiO_2_ solution. The addition of Fe^3+^ also has no influence on the fluorescent lifetimes. However, the absorption peak of FITC was greatly improved when Au_1_Ag_4_@SiO_2_ NPs were added (Fig. 7b). It is because that plasmonic metal NPs exhibit large absorption and scattering cross sections. So the fluorescence emission from one fluorescence molecule-nanoparticles hybrid nanostructures can be absorbed or scattered by the other hybrid NPs in the solution if the fluorescence emission peak overlaps with the plasmon resonance peak [2]. The fluorescence lifetime data and absorption spectra suggested that the electric field enhanced absorption is the dominant factor in our system. Moreover, the absorption of the FITC/Au_1_Ag_4_@SiO_2_ solution dramatically decreased after the addition of Fe^3+^ (Fig. 7b), indicating that the aggregation status of FITC molecules changed after the addition of Fe^3+^. The reason may be attributed to the complexation between isothiocyanate group and Fe^3+^. Isothiocyanate group of FITC can interact with the Fe^3+^ to form metal-isothiocyanate complexes [28]. When the Fe^3+^ was dropped into the FITC solution, bridges would be formed between FITC molecules and possibly induced FITC molecules to aggregate. The aggregation of FITC results in the FITC molecule being dropped away from the silica layer as well as the field enhancement region of NPs and the previously enhanced fluorescence of FITC by Au_1_Ag_4_@SiO_2_ NPs were greatly quenched again due to the promoted distance between FITC molecule and metal NPs.

## Conclusions

A facile and rapid approach for detecting low concentration of Fe^3+^ with improved sensitivity was developed through plasmon enhanced fluorescence and subsequently amplified fluorescent quenching. The fluorescence of the FITC solution was greatly improved by Au_1_Ag_4_@SiO_2_ due to the plasmon enhanced fluorescence. The enhanced fluorescence arises from surface plasmon enhanced absorption. However, efficient fluorescent quenching of the FITC solution was obtained when Fe^3+^ with a concentration ranging from 17 nM to 3.4 μM was further added into the FITC/Au_1_Ag_4_@SiO_2_ mixture, whereas no fluorescence quenching was observed for pure FITC solution. The amplified fluorescence quenching results in a great increase of sensitivity for detecting low concentration of metal ion. The quantized limit of detection (LOD) was improved from 4.6 μM for pure FITC solution to 20 nM for FITC/Au_1_Ag_4_@SiO_2_ solution. We believe this work would provide a general approach for preparing plasmonic chemsensor with high sensitivity for the detection of low concentration of metal ions.
